# Loss of Ewing sarcoma *EWS* allele promotes tumorigenesis by inducing chromosomal instability in zebrafish

**DOI:** 10.1038/srep32297

**Published:** 2016-08-25

**Authors:** Hyewon Park, Richard Galbraith, Thaddeus Turner, Justin Mehojah, Mizuki Azuma

**Affiliations:** 1 University of Kansas, Molecular Biosciences, Haworth Rm7031, 1200 Sunnyside Avenue, Lawrence KS 66045, USA; 2 Lawrence Memorial Hospital, Department of Pathology, 325 Maine St, Lawrence KS 66044, USA

## Abstract

The Ewing sarcoma family of tumors expresses aberrant *EWSR1- (EWS)* fusion genes that are derived from chromosomal translocation. Although these fusion genes are well characterized as transcription factors, their formation leaves a single *EWS* allele in the sarcoma cells, and the contribution that the loss of *EWS* makes towards disease pathogenesis is unknown. To address this question, we utilized zebrafish mutants for *ewsa* and *tp53*. The zebrafish *tp53(M214K)*^*w*/*m*^ line and the *ewsa*^*w*/*m*^, zygotic *ewsa*^*m*/*m*^, and Maternal-Zygotic (MZ) *ewsa*^*m*/*m*^ lines all displayed zero to low incidence of tumorigenesis. However, when the *ewsa* and *tp53* mutant lines were crossed with each other, the incidence of tumorigenesis drastically increased. Furthermore, 27 hour post fertilization (hpf) MZ *ewsa*^*m*/*m*^ mutant embryos displayed a higher incidence of aberrant chromosome numbers and mitotic dysfunction compared to wildtype zebrafish embryos. Consistent with this finding, tumor samples obtained from *ewsa*^*m*/*m*^;*tp53*^*w*/*m*^ zebrafish displayed loss of heterozygosity (LOH) for the wildtype *tp53* locus. These results suggest that wildtype Ewsa inhibits LOH induction, possibly by maintaining chromosomal stability. We propose that the loss of *ewsa* promotes tumorigenesis, and *EWS* deficiency may contribute to the pathogenesis of *EWS*-fusion-expressing sarcomas.

*EWS (Ewing sarcoma region 1, EWSR1)* was originally identified as part of a fusion gene with *FLI1,* in Ewing sarcoma cells^1^. Subsequent studies have shown that 90% of Ewing sarcoma tumors express the *EWS/FLI1* fusion gene; the remainder express *EWS*-fusions with other ETS transcription factors: *ERG*, *ETV1, ETV4,* and *FEV*^2^,^3^. In addition to *EWS-ETS* fusion genes, *EWS* is fused to other transcription factors in different sarcomas: *EWS/DDIT3* is expressed in myxoid liposarcoma, *EWS*/*ATF1* or *EWS*/*CREB* is expressed in clear cell sarcoma*, EWS*/*WT1* or *EWS*/*ERG* is expressed in desmoplastic round cell sarcoma, and *EWS*/*NR4A3* is expressed in extraskeletal myxoid chondrosarcoma^4^. In all cases, the amino terminal domain of EWS is preserved in the fusion protein, and the most well-characterized function of the hybrid protein is induction of aberrant transcription leading to epigenetic deregulation and altered expression of target genes^5^,^6^,^7^,^8^. However, loss of one *EWS* allele is also a consequence of fusion gene formation and has been observed in *EWS*-associated sarcomas. The functional significance of loss of *EWS* on the pathogenesis of *EWS*-fusion associated sarcomas is unknown.

EWS is a multifunctional protein that regulates transcription and splicing, and as a result, it affects numerous biological processes, including cell differentiation. An example of this is the role EWS plays in the differentiation of brown adipocytes, through activation of *BMP7* transcription^9^. EWS is also required for the differentiation of B cells^10^. Our previous study showed that EWS interacts with Sox9, a master regulator of chondrocyte differentiation, and promotes chondrogenesis by modulation of Sox9 transcriptional activity^11^. In addition, EWS regulates multiple phases of the cell cycle. This is demonstrated by the protein’s role in regulating the splicing of *CYCLIN D1* mRNA *in vitro*^12^,^13^. We previously reported that EWS maintains mitotic integrity^14^. We subsequently demonstrated that EWS interaction regulates the relocation of Aurora B kinase to the midzone during late mitosis^15^. Compromised relocation of Aurora B kinase results in chromosomal instability (CIN) and aneuploidy, both hallmarks of cancer^16^.

An animal model is a powerful approach for investigating the effect that loss of *EWS* has on transformation of normal cells into cancerous cells, because it enables the analysis of the molecular mechanism of transformation. The zebrafish is a well-established animal model for cancer research because of its suitability for live imaging, genetic analysis and drug screening^17^,^18^. Previously, we reported on two zebrafish *EWS* orthologues, *ewsa* and *ewsb,* and demonstrated that both zebrafish Ewsa and Ewsb regulate mitosis^14^. In this study, we utilized a zebrafish *ewsa* loss of function mutant to analyze the effect that *ewsa* mutation has on tumorigenesis. The zebrafish *tp53(M214K)* mutant, a well characterized cancer model, was utilized as a platform for studying tumor promotion. Berghmans, *et al*. reported that homozygous *tp53*^*M214K*/*M214K*^ (subsequently referred to as *tp53*^*m*/*m*^) mutant zebrafish display a 28% incidence of tumorigenesis by 16.5 mpf, whereas no tumors were reported in heterozygous *tp53*^*w*/*m*^ zebrafish^19^. In our study, we discovered that the zebrafish *ewsa* mutant promotes tumorigenesis in the *tp53*^*w*/*m*^ background by promoting loss of heterozygosity (LOH) of the wildtype *tp53* locus (that was accompanied by the loss of *tp53* wildtype allele), suggesting that Ewsa acts to suppress tumorigenesis. Furthermore, we demonstrate that Ewsa inhibits induction of CIN. Here, we propose a novel mechanism: loss of *EWS* contributes to pathogenesis in sarcomas expressing *EWS*-fusion genes.

## Results

### Loss of *ewsa* allele promotes tumorigenesis in *tp53*-mutation background zebrafish

**Figure 1:**
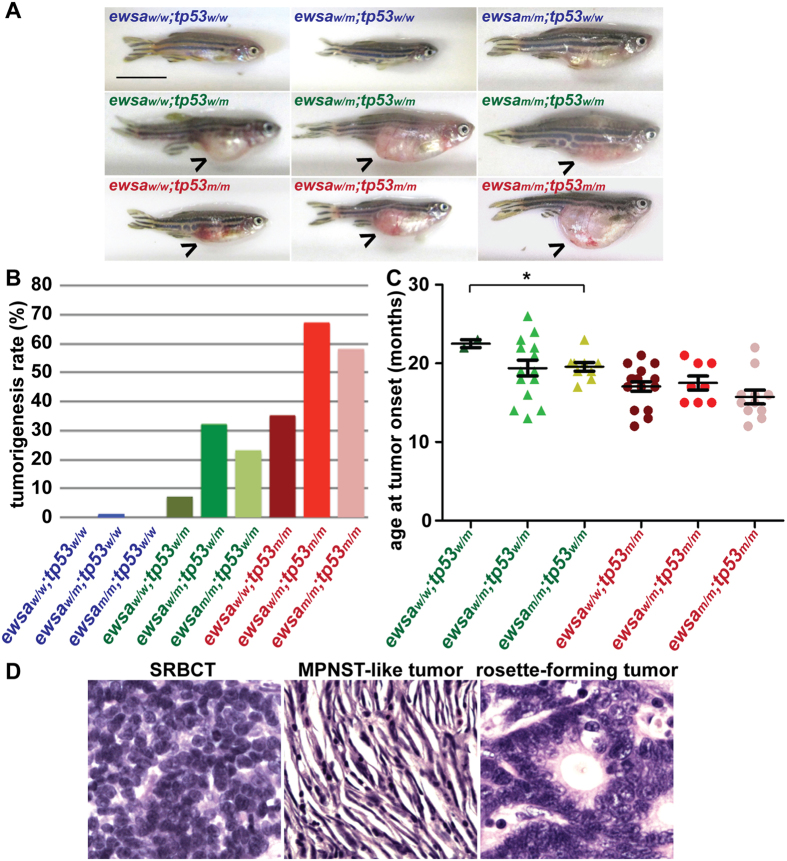
Loss of *ewsa* promotes tumorigenesis. Total numbers of fish for each genotypes are: *ewsa*^*w*/*w*^;*tp53*^*w*/*w*^ (n = 41), *ewsa*^*w*/*m*^;*tp53*^*w*/*w*^ (n = 135), *ewsa*^*m*/*m*^;*tp53*^*w*/w^ (n = 21), *ewsa*^*w*/*w*^;*tp53*^*w*/*m*^ (n = 28), *ewsa*^*w*/*m*^;*tp53*^*w*/*m*^ (n = 46), *ewsa*^*m*/*m*^;*tp53*^*w*/*m*^ (n = 39), *ewsa*^*w*/*w*^;*tp53*^*m*/*m*^ (n = 48), *ewsa*^*w*/*m*^;*tp53*^*m*/*m*^ (n = 12), *ewsa*^*m*/*m*^;*tp53*^*m*/m^ (n = 19). (**A**) Representative images of zebrafish. In images of *ewsa* and *tp53*mutants, tumors are indicated with an arrow (>). Scale bar: 1cm. (**B**) Tumor incidence is higher in *ewsa*^*w*/*m*^;*tp53*^*w*/*m*^ and *ewsa*^*m*/*m*^;*tp53*^*w*/*m*^ compared to *ewsa*^*w*/*w*^;*tp53*^*w*/*m*^; and in *ewsa*^*w*/*m*^;*tp53*^*m*/*m*^ and *ewsa*^*m*/*m*^;*tp53*^*m*/*m*^ compared to *ewsa*^*w*/*w*^;*tp53*^*m*/*m*^ zebrafish. (**C**) Average age at tumor onset is lower in *ewsa*^*w*/*m*^;*tp53*^*w*/*m*^ and *ewsa*^*m*/*m*^;*tp53*^*tp53w*/*m*^ compared to *ewsa*^*w*/*w*^;*tp53*^*w*/*m*^; and in *ewsa*^*w*/*m*^;*tp53*^*m*/*m*^ and *ewsa*^*m*/*m*^;*tp53*^*m*/*m*^ compared to *ewsa*^*w*/*w*^;*tp53*^*m*/*m*^ zebrafish. (**D**) Representative images of H&E staining of tumors observed in zebrafish. MPNST-like: malignant peripheral nerve sheath tumor-like; SRBCT: small round blue cell tumor.

Although EWS has an important role in regulating the cell cycle, and *EWS*-fusions in *EWS*-associated sarcomas result in the loss of one or both alleles of the gene, the contribution that this loss has on tumorigenesis is unknown. To address this question, we took advantage of an *ewsa* zebrafish mutant line that was originally generated using viral-insertion^11^. The insertion generated a premature stop codon at the seventh amino acid, creating a null mutant^11^. Heterozygous *ewsa*^*w*/*m*^ zebrafish from this line were incrossed to generate three genotypes (*ewsa*^*w*/*w*^, *ewsa*^*w*/*m*^, and *ewsa*^*m*/*m*^) that were subsequently monitored for visible signs of tumor formation or other signs of disease from 3 months post fertilization (mpf) (the age at which the fish reach sexual maturity) to 26 mpf. We did not observe any tumors in the *ewsa*^*w*/*w*^ (n = 41) or *ewsa*^*m*/*m*^ (n = 21) background, and only one fish with the *ewsa*^*w*/*m*^ genotype (n = 135) developed a tumor between 3 to 26 mpf. Because tumorigenesis is a complex process requiring multiple mutations, and because 10–15% of Ewing sarcoma patients have mutations in the tumor suppressor gene *TP53*, we chose to utilize the zebrafish *tp53* mutant to evaluate the synergistic effect of *ewsa* and *tp53* mutations. We generated fish with six genotypes: in the heterozygous *tp53*^*w*/*m*^ background*: ewsa*^*w*/*w*^;*tp53*^*w*/*m*^, *ewsa*^*w*/*m*^;*tp53*^*w*/*m*^, *ewsa*^*m*/*m*^;*tp53*^*w*/*m*^; and in the homozygous *tp53*^*m*/*m*^ background*: ewsa*^*w*/*w*^;*tp53*^*m*/*m*^, *ewsa*^*w*/*m*^;*tp53*^*m*/*m*^, and *ewsa*^*m*/*m*^;*tp53*^*m*/*m*^. The fish were monitored, and after 10 mpf, some began to develop visible tumor-like masses (Fig. 1A). These fish were subsequently euthanized and tumors were diagnosed by histological analysis using Hematoxylin and Eosin (H&E) staining of sectioned tissue. Consistent with the original report, few heterozygous *tp53*^*w*/*m*^ zebrafish with wildtype *ewsa* developed tumors; the incidence of tumorigenesis in *ewsa*^*w*/*w*^;*tp53*^*w*/*m*^ zebrafish was 8% after 26 months of observation (n = 28) (Fig. 1B). However, zebrafish with heterozygous or homozygous *ewsa* mutations in the heterozygous *tp53*^*w*/*m*^ background displayed a much higher tumor incidence. Zebrafish with the *ewsa*^*w*/*m*^;*tp53*^*w*/*m*^ genotype had a tumor incidence of 33% (n = 46) (Fig. 1B). Zebrafish with the *ewsa*^*m*/*m*^;*tp53*^*w*/*m*^ genotype displayed a tumorigenesis incidence of 23% (n = 39) (Fig. 1B). Heterozygous or homozygous *ewsa* mutations in the homozygous *tp53*^*m*/*m*^ background also increased tumor incidence. While the tumor incidence in zebrafish with the *ewsa*^*w*/*w*^;*tp53*^*m*/*m*^ genotype was 35% (n = 48), zebrafish with the *ewsa*^*w*/*m*^;*tp53*^*m*/*m*^ genotype had a tumor incidence of 67% (n = 12), and those with the *ewsa*^*m*/*m*^;*tp53*^*m*/*m*^ genotype had a tumor incidence of 58% (n = 19). These results are summarized in Table 1. The results suggest that loss of *ewsa* contributes to tumor formation, and Ewsa functions to suppress *tp53*-dependent tumorigenesis. Unexpectedly, tumor incidence was lower in homozygous *ewsa*^*m*/*m*^ zebrafish than in heterozygous *ewsa*^*w*/*m*^ zebrafish when expressed in either the heterozygous *tp53*^*w*/*m*^ or homozygous *tp53*^*m*/*m*^ backgrounds. One potential explanation for this is that cells with homozygous *ewsa*^*m*/*m*^ mutations have additional and unknown defects that result in cell death rather than transformation.

We also analyzed the age of tumor onset in fish from all genotypes. In the heterozygous *tp53*^*w*/*m*^ background, *ewsa*^*m*/*m*^;*tp53*^*w*/*m*^ zebrafish (20+/−2 months, total fish n = 39) had an significantly earlier age of tumor onset than *ewsa*^*w*/*w*^;*tp53*^*w*/*m*^ fish (23+/−1 months, n = 28) (P = 0.04). The *ewsa*^*w*/*m*^;*tp53*^*w*/*m*^ zebrafish (19+/−4 months, n = 46) also had earlier onset of tumorigenesis than *ewsa*^*w*/*w*^;*tp53*^*w*/*m*^ fish, but the difference was not significant. In the homozygous *tp53*^*m*/*m*^ background, zebrafish with homozygous *ewsa*^*m*/*m*^ mutations (16+/−3 months, n = 19) also displayed an earlier age of tumor onset than fish with wildtype *ewsa* (17+/−3 months, n = 48), but again, the difference was not significant. These results suggest that loss of *ewsa* contributes to tumor formation, and Ewsa suppresses tumorigenesis (Fig. 1C).

To address whether there is any tumor type specificity in the *ewsa* and *tp53* mutants, H&E stained tumor samples were characterized based on cell morphology. The tumors were subclassified into four types: 1. small round blue cell tumor (SRBCT); 2. malignant peripheral nerve sheath tumor (MPNST)-like tumors (which resemble the predominant tumors associated with *tp53* mutation as described in the original publication by Berghmans, *et al*.); 3. tumors with rosette pattern (rosette-forming tumors); and 4. other tumors (monitored fish displayed a low incidence of unclassified neoplasm, squamous cell carcinoma and melanoma) (Fig. 1D)^19^,^20^. No correlation was found between zebrafish genotype and tumor classification, including SRBCT and rosette-forming tumors that are tumor morphologies similar to those found in Ewing sarcoma (Table 1). These results suggest that the *ewsa* mutation does not specify cancer type. In summary, Ewsa has an inhibitory role in *tp53*-mutation dependent tumorigenesis, affecting both incidence and age at onset of tumor formation, but the *ewsa* mutation does not define the tumor type.

### Loss of *ewsa* allele promotes chromosomal instability (CIN)

**Figure 2:**
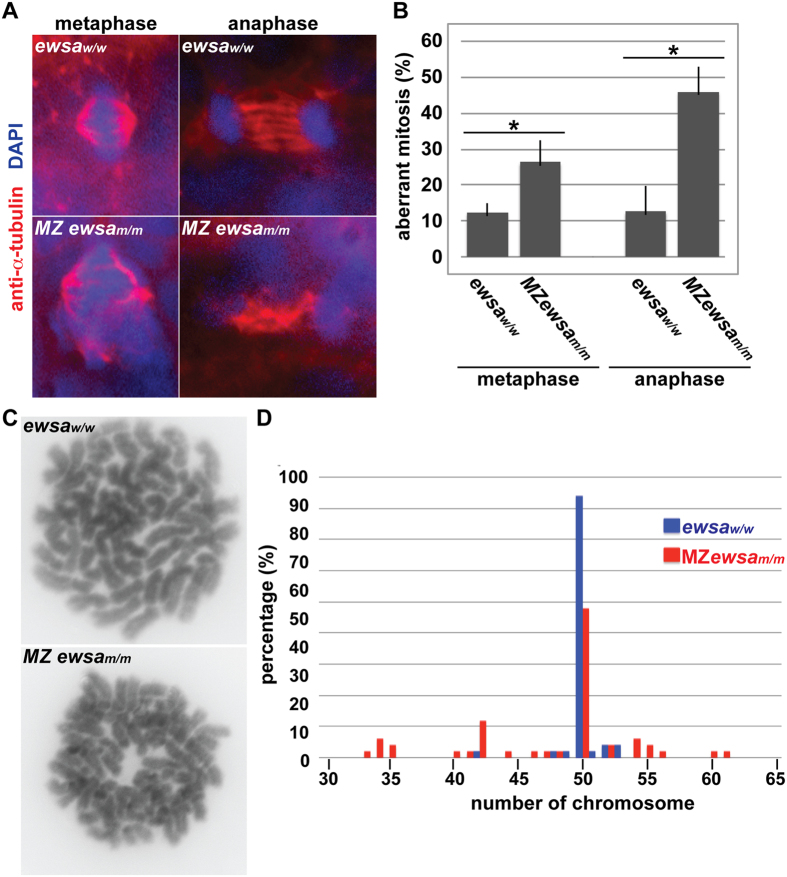
Loss of *ewsa* promotes chromosomal instability (CIN). (**A**) Representative images of mitotic cells from 27 hours post fertilization (hpf) zebrafish embryos visualized by immunohistochemistry using an anti-α-tubulin antibody (red) and DAPI (blue). (**B**) Percentages of cells with aberrant mitosis in zebrafish embryos at 27 hours post fertilization, as determined after immunohistochemistry using an anti-α-tubulin antibody. Experiments were repeated three times. (n = 13 to 60 mitosis from 3 to 6 embryos per genotype). *P < 0.05. (**C**) Representative images of chromosomes obtained from metaphase spread of 24 hpf *ewsa*^*w*/*w*^ (top) and MZ *ewsa*^*m*/*m*^ (bottom) embryos. (**D**) Numbers of chromosomes per cell, and percentage of cells from *ewsa*^*w*/*w*^ (blue bars) and MZ *ewsa*^*m*/*m*^ (red bars) zebrafish embryos containing that number of chromosomes. The vast majority of *ewsa*^*w*/*w*^ cells contained the normal chromosome number (50) (blue), whereas chromosome numbers in MZ *ewsa*^*m*/*m*^ mutant zebrafish cells varied from 34 to 56 chromosomes per cell (red). P < 0.05. Note that all samples were obtained from lines with wildtype *tp53*^*w*/*w*^ background.

Our previous studies showed that Ewsa in zebrafish, or EWS in human cell lines, regulates midzone formation during mitosis^14^,^15^. The structure known as the midzone forms midline consisted of central spindles between segregating chromosomes during anaphase^21^,^22^,^23^. Aberrant midzone formation leads to failure of cell division, and induces aberrant chromosome distribution to daughter cells, in an event known as chromosomal instability (CIN)^24^. To address whether Ewsa regulates central spindle formation, maternal and zygotic *ewsa* mRNA was depleted from zebrafish embryos by incrossing homozygotic *ewsa*^*m*/m^ mutant zebrafish (Maternal-Zygotic (MZ) *ewsa*^*m*/*m*^ zebrafish). Immunohistochemistry using anti-α-tubulin antibody was performed on 27 hour post-fertilization (hpf) *ewsa*^*w*/*w*^ and MZ *ewsa*^*m*/*m*^ zebrafish. Mitosis was visualized in both wildtype and MZ *ewsa*^*m*/*m*^ embryos and the numbers of aberrant mitotic events were scored. Consistent with our previous results in knockdown experiments, (using morpholinos designed against *ewsa* mRNA), the MZ *ewsa*^*m*/*m*^ displayed a higher incidence of mitotic dysfunction compared to *ewsa*^*w*/*w*^ (Fig. 2A,B)^14^. Specifically, the incidence of spindle defects at anaphase and at metaphase was higher in cells from MZ *ewsa*^*m*/*m*^ embryos than in *ewsa*^*w*/*w*^. The result suggests that Ewsa regulates mitosis, particularly central spindle formation.

It is known that mitotic dysfunction leads to uneven distribution of chromosomes between daughter cells. Therefore, chromosome numbers were quantified in cells obtained from 24 hpf *ewsa*^*w*/*w*^ and MZ *ewsa*^*m*/*m*^ embryos, using metaphase spreads (Fig. 2C). The majority of cells obtained from *ewsa*^*w*/*w*^ embryos displayed the normal number of chromosomes (50 chromosomes). However, the MZ *ewsa*^*m*/*m*^ mutants displayed a higher percentage of cells with aberrant chromosome numbers compared to wildtype zebrafish (Fig. 2D). Together with the evidence for defective central spindle formation in cells from MZ *ewsa*^*m*/*m*^ embryos, these results suggest that Ewsa maintains chromosomal stability. Interestingly, despite the results that show a large percentage of cells with abnormal chromosome numbers at 27 hpf in MZ *ewsa*^*m*/*m*^ mutants, these embryos survive to adulthood and the adults are fertile. How the embryo survives with this level of aneuploidy is unclear. One potential explanation is that the aneuploidy observed at 27 hpf is the result of CIN in a cell type that has less impact on the embryo’s survival, possibly due to a low proliferation rate.

### Loss of *ewsa* allele promotes Loss of Heterozygosity (LOH) of *tp53* in tumor cells

**Figure 3:**
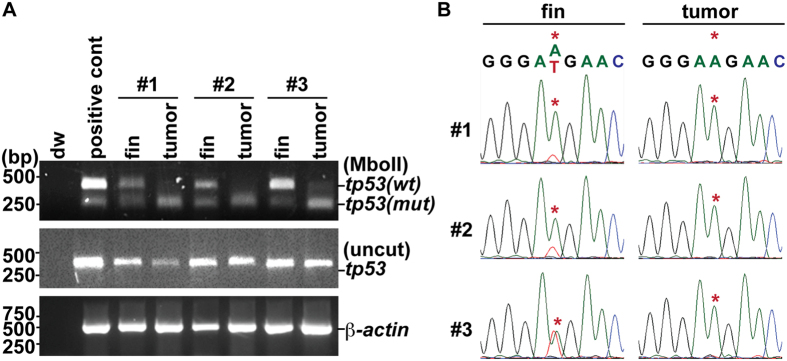
Loss of *ewsa* promotes LOH of *tp53* locus. (**A**) RFLP analysis of *tp53* locus obtained from DNA from fin (with no tumor), and tumor samples. MboII digests the *tp53* mutation allele, but not the *tp53* wildtype allele. #1–3: each number represents individual fish. Positive cont: genomic DNA obtained from a fin from a heterozygous *tp53*^*w*/*m*^ fish. wt: *tp53* wildtype allele; mut: *tp53* mutant allele. (**B**) Sequence trace of *tp53* locus obtained from fin and tumor samples of fish #1–3. The point mutation site of *tp53* allele is indicated with (*).

Homozygous *tp53*^*m*/*m*^ zebrafish displayed a high incidence of tumors (35%, n = 48), while zebrafish with the heterozygous *tp53*^*w*/*m*^ genotype showed a low incidence of tumor development (8%, n = 28) over 26 mpf (Table 1)^19^. However, when we generated heterozygous *tp53*^*w*/*m*^ zebrafish with homozygous *ewsa*^*m*/*m*^ mutations, we observed a significant increase in tumor incidence (23%, n = 39) (Table 1). We hypothesized that loss of *ewsa* may promote tumorigenesis by inducing LOH of the *tp53* wildtype locus. To investigate LOH, we collected tumors obtained from three individual fish (that had been genotyped prior to tumor formation and identified as *ewsa*^*m*/*m*^;*tp53*^*w*/*m*^ from fin (non-tumor) DNA). The genotypes of DNA extracted from fins and tumors were assayed using Restriction Fragment Length Polymorphism (RFLP)^19^. The restriction enzyme MboII digests PCR products amplified from the *tp53* mutation allele, but not from the *tp53* wildtype allele^19^. This analysis demonstrated that all three fins were the heterozygous *tp53*^*w*/*m*^ genotype. Conversely, RFLP analysis of the tumors demonstrated loss of the *tp53* wildtype allele, suggesting that the tumors underwent LOH for *tp53* (Fig. 3A). LOH of the *tp53* locus was verified by sequencing of the PCR product containing the mutation site (*M214K)*. Sequencing verified that all three fin samples were *tp53*^*w*/*m*^, whereas all tumors lost the *tp53* wildtype allele (Fig. 3B). From this data, it is unknown whether the *tp53* mutant locus underwent duplication, or whether the *tp53* wildtype locus was deleted. However, we conclude that loss of *ewsa* leads to induction of *tp53* LOH with loss of the *tp53* wildtype locus. Our study suggests a novel mechanism for the pathogenesis of *EWS* fusion-associated sarcomas: loss of one *EWS* allele due to the formation of the *EWS*-fusion gene induces mitotic dysfunction accompanied by aberrant midzone formation, CIN, and promotion of tumorigenesis by inducing LOH. Loss of the *ewsa* allele did not change the tumor type that developed (Table 1). Thus, it is likely that both loss of *EWS* and *EWS*-fusion expression are required for promotion of tumorigenesis, and determination of tumor type.

## Discussion

Formation of *EWS*-fusion genes in Ewing sarcoma results in the loss of one or both wildtype *EWS* alleles in the sarcoma cells^2^,^25^. While most studies involving Ewing sarcoma have focused on the function of the *EWS*-fusion genes, there is little understanding of the role that loss of an *EWS* allele has on pathogenesis. To address this, use of an animal model is essential; therefore, we utilized the *ewsa* zebrafish mutant. Although *ewsa*^*w*/*m*^ and *ewsa*^*m*/*m*^ mutant zebrafish have no or a low incidence of tumors, heterozygous or homozygous *ewsa* mutation accelerated tumorigenesis in the *tp53*^*w*/*m*^ background. This result suggests that the loss of one or both alleles of *ewsa* promotes tumorigenesis. We also discovered that mutation of both *ewsa* alleles results in CIN. Consistent with this discovery, tumors that developed in *ewsa*^*m*/*m*^;*tp53*^*w*/*m*^ zebrafish underwent LOH for the wildtype *tp53* allele. This is the first demonstration in an animal model of the role of Ewsa in the maintenance of chromosomal stability, and in suppressing tumorigenesis. Establishing double mutants for *ewsa* and *tp53* in zebrafish allowed us to compare large sample numbers of each genotype. These zebrafish lines will serve as a useful model in future studies to investigate the contribution of Ewsa haploinsufficiency towards the pathogenesis of *EWS*-fusion associated sarcomas.

Elucidating the mechanism of Ewsa-dependent LOH induction is another critical step in understanding the pathogenesis of Ewing sarcoma tumors. Our previous study demonstrated that zebrafish *ewsa* knockdown induces chromosomal mis-segregation due to mitotic dysfunction. Chromosome exchange occurs during metaphase when sister chromatids are aligned. Because *EWS* knockdown alters the localization of Aurora B kinase during anaphase and metaphase, this may disrupt the critical orchestration of chromosome segregation, leading to induction of LOH^15^. Another possible explanation for LOH induction is that loss of *ewsa* mitotic function leads to DNA damage, and DNA repair failure contributes to LOH. Previous studies have shown that *EWS* knockdown results in alternative splicing of DNA repair molecules (e.g. *ABL1*, *CHEK2* and *MAPK2*). Therefore, it is possible that LOH is induced by compromising two separate functions of EWS: mitosis and splicing of DNA repair molecules. In this study, we utilized *tp53* zebrafish as a tool to analyze tumorigenesis, but it is likely that other tumor suppressor gene loci undergo LOH as well. Recent studies have shown the existence of common fragile sites that are regions of genomic instability and thus are “hot-spots” for recombination^26^,^27^. These regions are enticing candidates for study to determine whether Ewsa/EWS regulates these loci to suppress tumorigenesis. In addition, it is also possible that the *tp53* wildtype locus was deleted due to the induction of CIN.

Mesenchymal stem cells are considered to be the cell of origin in Ewing sarcoma^28^,^29^. Interestingly, most other *EWS*-fusion associated sarcomas develop in tissues that derive from mesenchymal cells. For this reason, the mesenchymal stem cell is a strong candidate to be the cell of origin for other *EWS*-fusion associated tumors. Our zebrafish model will allow us to investigate the cell of origin for *EWS* associated tumors, in a spatiotemporal manner, because both developmental and differentiation processes in the mesenchymal cell lineage are conserved between zebrafish and human. In addition, because the frequency of LOH induction may be increased in the tumor cell of origin, our zebrafish model will allow us to measure the frequency of LOH induction in various cell types, and this may provide a platform to elucidate the mechanism of LOH.

Finally, studies in which *EWS/FLI1* was overexpressed in animal models suggest that expression of *EWS/FLI1* alone is not sufficient to induce Ewing sarcoma. To achieve conditional expression of *EWS/FLI1* in a mouse model, *EWS/FLI1* transgenic mice were crossed to *Prx1-Cre* transgenic mice^30^. The mice from these crosses did not develop tumors, but *EWS/FLI1* did accelerate the formation of sarcomas in *Tp53* deleted mice^30^. Zebrafish with mosaic expression of human *EWS/FLI1* had a very low incidence of tumor formation, but crossing these with a *tp53* mutant increased the incidence of tumorigenesis^20^. In addition, when *EWS/FLI1-*transduced mesenchymal stem cells were injected into mice, they developed fibrosarcoma^31^. Together, these findings suggest that the pathogenesis of Ewing sarcoma may require both the expression of the *EWS*-fusion gene and the loss of an *EWS* allele. This hypothesis can be expanded to other *EWS*-associated sarcomas as well, as loss of one wildtype allele is a common feature to all. In addition, we previously reported that expression of EWS/FLI1 leads to mitotic defects through dominant inhibition of EWS^14^,^32^. Previous studies have shown that EWS/FLI1 and EWS biochemically interact^32^,^33^,^34^. This interaction may further deplete the normal function of the remaining wildtype *EWS* allele. We previously demonstrated that EWS relocates the mitotic regulator Aurora B kinase to the midzone^15^. The midzone is required for the maintenance of spindle architecture, spindle elongation, and cleavage furrow formation^23^,^35^. Failure of Aurora B kinase to relocate to the midzone leads to defects in cytokinesis accompanied by uneven chromosome segregation, and the induction of aneuploidy^36^. Therefore, it is possible that the high incidence of aberrant chromosome numbers observed in *ewsa* mutant zebrafish may be the result of mitotic defects due to the loss of Ewsa. All of this suggests that *EWS* haploinsufficiency may play a critical role in the tumorigenesis of *EWS*-fusion associated sarcomas.

## Materials and Methods

### Ethics statement

All protocols utilizing zebrafish were approved by the University of Kansas, Lawrence, Institutional Animal Care and Use Committee (IACUC) (Animal use statement: permit number #197–02). All methods were carried out in accordance with the relevant guidelines.

### Zebrafish maintenance

The *ewsa* zebrafish mutant line was established and characterized in a previous report^11^. The Oregon AB, *ewsa,* and *tp53*^*M214K*^ zebrafish mutant lines were housed at 28 °C in an automated filtration system from Aquatic Habitats.

### Histological analysis

Zebrafish were humanely euthanized, and tissues fixed with 4% paraformaldehyde at 4 °C overnight. The fixed samples were embedded, sectioned, and subjected to Hematoxilin and Eosin (H&E) staining using standard protocols.

### Immunohistochemistry

Zebrafish 27 hpf embryos were fixed with 4% paraformaldehyde at 4 °C overnight, and subjected to immunohistochemistry using anti-α-tubulin antibody (Sigma) as described previously^14^.

### Metaphase chromosome spreads

Chorionic membranes from 24 hpf zebrafish embryos were removed. The embryos were euthanized with Tricaine mesylate, and rinsed twice in PBS. The embryos were then placed in 1.1% NaCitrate at room temperature for 8 min. The embryonic cells were dissociated using a syringe, and placed on ice for an additional 8 min, then were collected by centrifugation (3000 rpm for 5 min). The supernatant was replaced with methanol:acetic acid (3:1) and cells were incubated for 20 min at room temperature, and subsequently incubated at −20 °C overnight. The cells were collected by centrifugation, resuspended in methanol:acetic acid (3:1) solution, and this suspension was dropped onto glass slides and the DNA was counterstained with DAPI.

### Statistics

Error bars represents standard deviation (SD), and statistical confidence was determined to be at P < 0.05 by t-test (Figs 1C and 2B), or t-tests for normal distribution (Fig. 2D).

## Additional Information

**How to cite this article**: Park, H. *et al*. Loss of Ewing sarcoma *EWS* allele promotes tumorigenesis by inducing chromosomal instability in zebrafish. *Sci. Rep.* **6**, 32297; doi: 10.1038/srep32297 (2016).

## Figures and Tables

**Table 1:** Percentages of tumor type for each genotype.

genotype	*ewsa*^*w*/*w*^ *tp53*^*w*/*w*^	*ewsa*^*w*/*m*^ *tp53*^*w*/*w*^	*ewsa*^*m*/*m*^ *tp53*^*w*/*w*^	*ewsa*^*w*/*w*^ *tp53*^*w*/*m*^	*ewsa*^*w*/*m*^ *tp53*^*w*/*m*^	*ewsa*^*m*/*m*^ *tp53*^*w*/*m*^	*ewsa*^*w*/*w*^ *tp53*^*m*/*m*^	*ewsa*^*w*/*m*^ *tp53*^*m*/*m*^	*ewsa*^*m*/*m*^ *tp53*^*m*/*m*^
MPNST-like (%)	0	0	0	4	9	10	10	42	26
SRBCT (%)	0	0.5	0	4	20	13	10	25	27
Rosette (%)	0	0.5	0	0	0	0	9	0	5
Others (%)	0	1	0	0	4	0	6	0	0
Total (%)	0	1	0	8	33	23	35	67	58
Tumor (n)	0	1	0	2	15	9	17	8	11
Total fish (n)	41	135	21	28	46	39	48	12	19

MPNST-like: malignant peripheral nerve sheath tumor-like; SRBCT: small round blue cell tumor.
